# Sex-related differences in incidence, phenotype and risk of sudden cardiac death in inherited arrhythmia syndromes

**DOI:** 10.3389/fcvm.2022.1010748

**Published:** 2023-01-04

**Authors:** Babken Asatryan, Andreas S. Barth

**Affiliations:** ^1^Department of Cardiology, Inselspital, Bern University Hospital, University of Bern, Bern, Switzerland; ^2^Division of Cardiology, Department of Medicine, Johns Hopkins University School of Medicine, Baltimore, MD, United States

**Keywords:** estrogen, progesterone, testosterone, long QT syndrome, Brugada syndrome, catecholaminergic polymorphic ventricular tachycardia, short QT syndrome, precision medicine

## Abstract

Inherited Arrhythmia Syndromes (IAS) including long QT and Brugada Syndrome, are characterized by life-threatening arrhythmias in the absence of apparent structural heart disease and are caused by pathogenic variants in genes encoding cardiac ion channels or associated proteins. Studies of large pedigrees of families affected by IAS have demonstrated incomplete penetrance and variable expressivity. Biological sex is one of several factors that have been recognized to modulate disease severity in IAS. There is a growing body of evidence linking sex hormones to the susceptibility to arrhythmias, yet, many sex-specific disease aspects remain underrecognized as female sex and women with IAS are underinvestigated and findings from male-predominant cohorts are often generalized to both sexes with minimal to no consideration of relevant sex-associated differences in prevalence, disease manifestations and outcome. In this review, we highlight current knowledge of sex-related biological differences in normal cardiac electrophysiology and sex-associated factors that influence IAS phenotypes.

## 1. Introduction: The significance of sex differences in inherited cardiac arrhythmias

**Figure 1:**
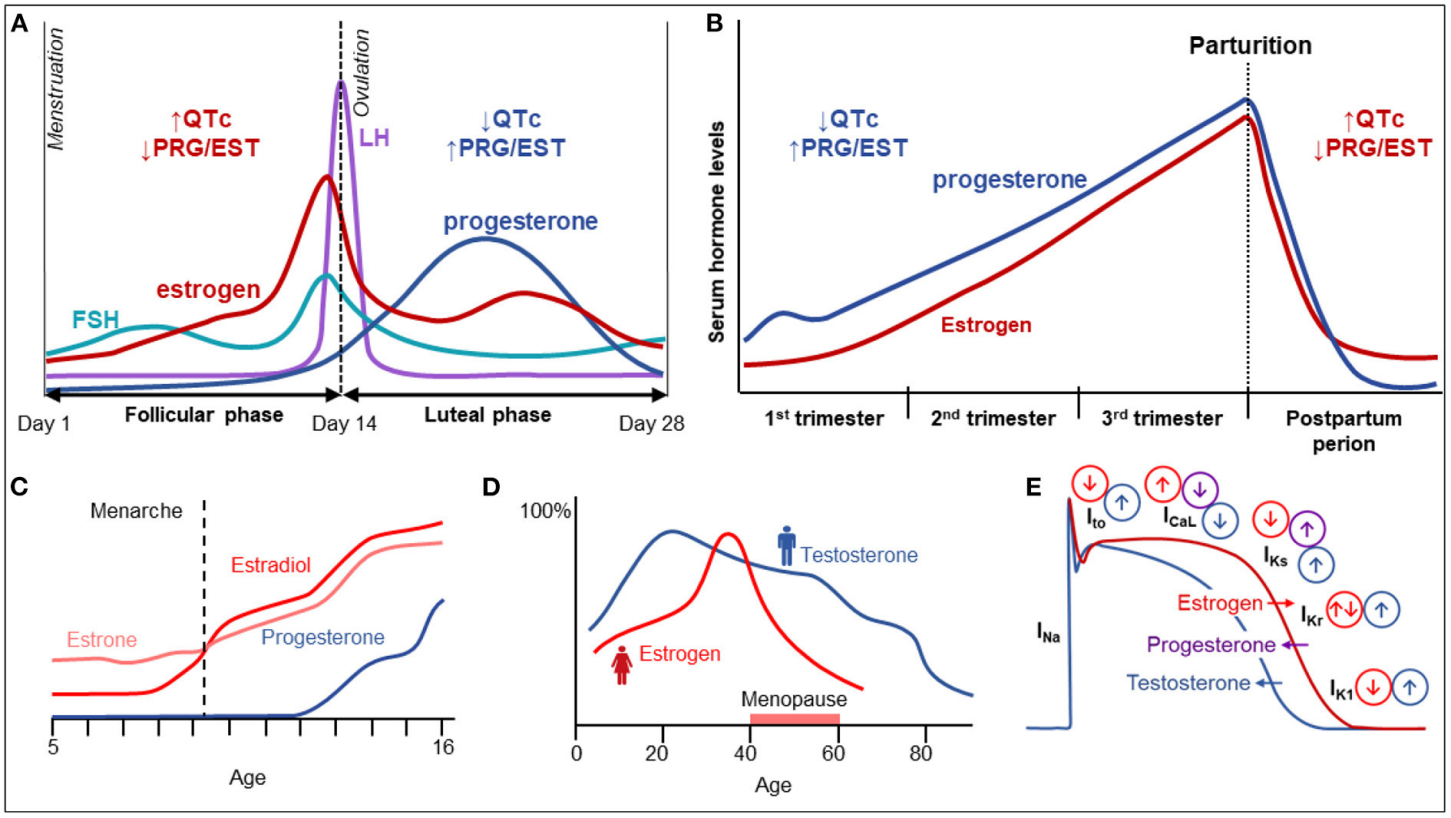
Sex hormones and their influence on cardiac electrophysiology. **(A)** Variation in female hormone levels during the menstrual cycle. **(B)** Variation in female sex hormones and electrical parameters during pregnancy and the postpartum period. **(C)** The increase of female sex hormones until adolescence. **(D)** The variation of female and male sex hormones with age. **(E)** The influence of various hormones on cardiac electrical currents and action potential duration.

Inherited Arrhythmia Syndromes (IAS) can lead to sudden cardiac death (SCD) in the absence of apparent structural heart disease and are caused by pathogenic variants in genes encoding cardiac ion channels or associated proteins. Studies of large pedigrees of families affected by IAS have demonstrated incomplete penetrance and variable expressivity. Biological sex is one of several factors that have been recognized to modulate disease severity in IAS *via* the effect of steroid and non-steroid hormones on cardiac ion channels (1). In women, the risk of arrhythmias varies during different phases of the menstrual cycle, influenced by shifts in the balance of the steroid hormones estrogen and progesterone, while in men, testosterone has been shown to regulate expression of critical myocardial ion channels (Figure 1). Herein, we will review evidence from basic research and clinical studies linking sex hormones to the susceptibility to arrhythmias in IAS. A better understanding of the role of sex-related differences in modulating clinical outcomes in IAS will lead to improvement in individualized risk prediction of SCD and clinical management of patients with IAS.

## 2. Sex differences in cardiac electrophysiology

Biological sex, among other factors, has been recognized as a modifier of cardiac electrical activity. In this respect, genetic differences and variations in sex hormones play an important role, while other aspects such as autonomic tone, hemodynamics, and non-steroid hormone levels may also contribute to sex-related differences but their roles are insufficiently studied. While males tend to have longer PR intervals, P-wave and QRS-durations, adult women have been shown to higher resting heart rate as well as longer heart-rate corrected QT intervals (QTc) (2). With the onset of puberty, males manifest QT shortening while females develop QT prolongation. Additionally, these apparent differences in QT intervals diminish in postmenopausal women, suggesting a prominent role of sex hormones in modulation of the QT interval (2–4). During the menstrual cycle, shorter QTc durations are observed in the luteal phase, considered an effect of higher progesterone levels (5). Furthermore, as the QT/RR slope is steeper in women, differences in QTc duration are more manifest at slow heart rates (6).

## 3. Long QT syndrome

Congenital Long QT Syndrome (LQTS) is a primary electrical disorder characterized by a prolonged repolarization phase of the cardiac action potential, reflected by a prolonged QT interval on the surface electrocardiogram (ECG). Clinically, LQTS leads to a predisposition to arrhythmias, including polymorphic ventricular tachycardia (torsades de pointes, TdP) and sudden cardiac death (7). Over the past 25 years, 17 genes have been reported in association with LQTS; however, a recent literature curation by an international expert group found only 6 genes –*KCNQ1, KCNH2, SCN5A*, and *CALM1-3* to have definitive evidence for causing LQTS (8). Variants in the LQT1-3 genes (*KCNQ1, KCNH2* and *SCN5A*, respectively) are responsible for ≈90% of all reported genotype-positive cases (9).

As significant sex-related differences in the QT-interval exist, sex is considered an important factor in the management of LQTS patients (Table 1). QT intervals are generally longer in healthy women as compared to men; therefore, different cut-offs for prolonged QTc have been established−470 ms in men and 480 ms in women (10). Among patients with disease-causing variants in LQTS-associated genes, women have higher disease penetrance than men (11), and comprise around 56% of all LQTS cases (12, 13). Women with LQT1 and LQT2 have longer QTc than affected men (14). Women are more susceptible to developing prolongation of the QT at slower heart rates, making QTc duration at rest and during sleep critical markers of arrhythmic risk (14). The risk of TdP also varies during the lifespan with adult women with LQTS being at higher risk of TdP than males and pre-pubertal females (15).

**Table 1:** Summary of sex-related differences in inherited arrhythmia syndromes.

**IAS**	**Relevant sex-related differences**	**Reference(s)**
LQTS^*^	Among patients with disease-causing LQTS gene variants, women have higher disease penetrance than men. Women with LQT1 and LQT2 have longer QTc than affected men.	(11, 14, 15, 17, 19)
	Women are more susceptible to developing prolongation of the QT at slower heart rates.	
	Adult women with LQTS have higher risk of TdP compared to males and females of pre-pubescent age.	
	Men with LQTS have a higher risk of fatal presentation, while women often have recurrent syncopal episodes.	
	In childhood and prior to puberty, males with LQT1 have an increased risk for arrhythmias compared to females, but the arrhythmic risk reverses after puberty with higher risk in females.	
	The risk of arrhythmias is also higher in post-pubertal women with LQT2 compared to men, and it stays elevated at post-reproductive age.	
	Among patients with LQT3, females in general, and particularly at 30–40 years age, are at higher risk for SCD. Women are more susceptible to developing drug-induced LQTS and can manifest TdP at relatively shorter QTc intervals.	
BrS	BrS phenotype is identified 8–10 times more frequently frequently in men, particularly in Southeast Asia.	(45–49, 59)
	Women with BrS are more frequently asymptomatic at the time of diagnosis, and 6–7 years older than men both at the time of diagnosis (49 vs. 43 years) and at the time of the first arrhythmic event (50 vs. 43 years).	
	Female BrS patients less frequently have a spontaneous type 1 Brugada ECG pattern (22–41 vs. 36–69%) or ventricular arrhythmia inducibility at electrophysiology study (27–36 vs. 42–66%).	
	Women are 3–4 times less likely to experience arrhythmic events, i.e., syncope, aborted cardiac arrest and documented VF, than men with BrS, except in the pediatric age group, where a spontaneous BrS ECG is associated with earlier onset of arrhythmic events.	
	Fragmented QRS and QRS prolongation (>120 ms) are important risk factors for arrhythmic events in women with BrS (HR 20.2 and 4.7, respectively).	
	Men with Brugada syndrome have been shown to have a larger arrhythmogenic substrate as compared to women.	
CPVT	The penetrance is high in both sexes and the risk of tachyarrhythmias appears to be mostly variant type/location dependent.	(68)
	The Canadian founder *RyR2*-p.R420W variant showed earlier mortality in affected men compared to women.	
SQTS	Nearly 70% of all patients diagnosed with SQTS are males, suggesting a sex-dependent penetrance.	(75, 76)
	No sex-related difference in QTc duration among those diagnosed with SQTS have been identified.	
	Affected men have a 3-fold higher risk of syncope at first presentation with a similar risk of SCD compared to females (24 vs. 25%).	
	No conclusions can be made regarding the risk of tachyarrhythmias as the available studies mostly include incomparable number of men and women with ICDs, which understandably results in differences in the VT/VF detection rates.	
ER pattern and ERS	The ER pattern is more often found in young men (>70% of cases) than in women, with reduced prevalence and diminished sex differences with increasing age.	(79–81)
	The ER pattern is more common in adolescents and athletes. In African Americans, an ER pattern has a prevalence of up to 25%, and has no association with tachyarrhythmias or SCD. Ethnicity may contribute to a higher arrhythmogenic risk with Caucasians being more susceptible.	
	Sex-differences in terms of risk have not been reported, but ischemia is known to provoke more arrhythmias in patients with ER pattern, and since men are more susceptible to coronary ischemia, they might be more susceptible to ER-related arrhythmias.	
IVF	Occurs in presumably healthy middle-aged individuals of either sex	(101–104)
	According to a meta-analysis of 23 studies, males comprise about 70% of all IVF patients.	
	Male sex, younger age, and presence of symptoms preceding the index event seem to be associated with multiple appropriate ICD shocks.	
	Affected men are more likely to receive a specific diagnosis during follow-up.	
	Among carriers of the DPP6 haplotype, men show higher rates of VF and lower survival than women (63 vs. 83 years).	
PCCD	No sex-related differences have been described.	(108)
	As in other *SCN5A*-mediated diseases, PCCD seems to be more common among men, but robust data is lacking.	
	Data on risk during different reproductive phases does not exist.	
Familial ST-depression syndrome	While equal predisposition to the syndrome among men and women is expected given its autosomal dominant inheritance pattern, male patients appear to be more likely to develop AF or (aborted) SCD.	(110)
	The occurrence of left-ventricular systolic dysfunction is almost exclusively observed in men.	

BrS, Brugada syndrome; CPVT, catecholaminergic polymorphic ventricular tachycardia; ER, early repolarization; ERS, early repolarization syndrome; IAS, inherited arrhythmia syndrome; LQTS, long QT-syndrome; PCCD, progressive cardiac conduction disease; SCD, sudden cardiac death; SQTS, short QT-syndrome; TdP, torsades de pointes; VF, ventricular fibrillation; VT, ventricular tachycardia.

* Details regarding genotype-specific differences in phenotype and management are provided in the “Long QT syndrome” section of the manuscript.

In contrast, men with LQTS have a higher risk of a fatal presentation, while women often have recurrent, non-fatal events, such as syncopal episodes (11). Men suffering from LQT1 and LQT2 have clinical manifestations at a younger age than affected women (16). In childhood and prior to puberty, boys with LQT1 (but not LQT2 or LQT3) are at a higher risk for arrhythmic manifestations than girls (17), but the arrhythmic risk reverses after puberty with higher risk in females (11). The risk of arrhythmias is also higher in post-pubertal women with LQT2 compared to men (17), and it stays elevated at post-reproductive age (18). Among patients with LQT3, females in general, and particularly at 30–40 years age, are at higher risk for SCD (19).

### 3.1. Acquired long QT syndrome

Pathogenic variants in *KCNE1* and *KCNE2* genes have been reported in association with drug or electrolyte-provoked LQTS, referred to as acquired LQTS; certain *KCNE1* variants have also been implicated in congenital LQTS (8). Risk factors for drug-induced LQTS include underlying bradyarrhythmias or abrupt heart rate slowing (the “short–long–short” cycle length changes), female sex, hypokalemia and hypomagnesemia, the presence of advanced underlying structural heart disease, recent conversion from atrial fibrillation, previously unrecognized congenital LQTS, and polypharmacy, particularly, use of multiple QT-prolonging drugs (20, 21). Clinical studies have shown that female sex is associated with a 2–3-fold higher risk of developing drug-induced QT prolongation for both cardiovascular (22) and non-cardiovascular medications (23). This is also supported by the findings that females are prone to TdP at relatively shorter QTc intervals than their male counterparts (24). This higher susceptibility to drug-provoked QT prolongation is not fully explained by differences in plasma levels of sex hormones, but rather is attributed in part to female intrinsic sensitivity (25).

### 3.2. Clinical implications

β-blockers are the therapeutic cornerstone in congenital LQTS and recommended in both sexes (26). Therapy with β-blockers (preferably a non-selective beta blocker like nadolol) is most effective in LQT1, but also shows up to 70% efficacy in LQT2 (17). Response to β-blockers varies by sex and underlying genotype so that adult LQT1 men benefit most from β-blockade (27). Asymptomatic pre-adolescent boys with a QTc >500 ms are at >12-fold increased risk of life-threatening cardiac arrhythmias as compared to their female peers, highlighting the urgent need of therapy in these patients (28). Selected asymptomatic adult men with LQTS, particularly those with older age at diagnosis and QTc <470 ms, have the lowest arrhythmic risk, but may still benefit from low dose β-blocker therapy (11, 29), while the risk in females might be increased after puberty due to the influence of sex hormone on cardiac repolarization, requiring escalation of β-blocker therapy.

The evaluation of therapy in LQT3 is more difficult since these cases are less common. In one study, β-blockers resulted in 83% reduction in arrhythmic events in women but not in men with LQT3; men, however, had significantly fewer events (19). Patients with LQT3 and poor adherence to therapy with β-blockers may benefit from a left cardiac sympathetic denervation (LCSD) (30). In selected LQT3 patients, who remain symptomatic or have a QTc > 500 ms notwithstanding therapy with β-blockers, mexiletine can be considered (31, 32). Alternatively, flecainide may be used in LQT3, in the absence of flecainide-provoked Brugada ECG pattern, that is more frequently seen in males (33). Over the past years, the indications for ICD implantation have been revisited and currently only a small number of LQTS patients are eligible for ICD insertion; these primarily include survivors of cardiac arrest and patients at very high-risk for SCD who have recurrent syncope despite adequate therapy with β-blockers (26).

K^+^ supplementation is a rational additional therapy in all LQTS patients and may be especially useful in LQT2 patients. Potassium-sparing diuretics may be used as an add-on therapy in patients with significant and frequent hypokalemia.

In women with LQTS, the probable QTc-prolonging effect of synthetic progesterone should be taken into account when making decisions regarding contraception (34). A recent retrospective study by Goldenberg et al. evaluated the arrhythmic risk of three types of oral contraceptives (progestin-only, estrogen-only, and the combination of progestin and estrogen) in 370 women with LQTS. In this study, progestin-only therapy was associated with an increased risk of arrhythmic events in LQTS (35), particularly in the absence of concomitant β-blocker therapy. Additionally, the authors found that LQT2 female patients had an increased risk of cardiac events when on oral contraceptives as compared with other LQTS genotypes, suggesting that oral contraceptives should be used with caution in LQT2 women without concomitant β-blocker therapy (35).

Men with LQTS should be evaluated for low serum testosterone levels, androgen deprivation therapy exposure, and endocrine disorders associated with hypogonadism, since these factors have been associated with higher risk for drug-induced TdP (36, 37), and might represent modifiable risk factors in men with congenital LQTS. A recent small placebo-controlled study showed that transdermal testosterone attenuates the QT-prolonging effects of ibutilide in older men, suggesting that androgens might be useful to prevent or treat TdP in men with drug-induced LQTS (38). The applicability of these findings to congenital LQTS is unclear.

### 3.3. Pregnancy

Given the significant changes of sex hormones during pregnancy and the post-partum period, it is important to consider their modifier effect on the LQTS phenotype. Females, particularly those with LQT1, are at a lower risk for arrhythmias during the course of pregnancy (39). Arrhythmic risk is increased in the early post-partum period, particularly in patients with LQT2 (39) prior to returning to the pre-pregnancy baseline (39). β-blockers are effective in reducing the risk of arrhythmic events during pregnancy and are particularly essential at the post-partum period to prevent life-threatening manifestations (39). Non-selective β-blockers are preferred for women in the post-partum period, but metoprolol is the most studied in terms of fetal safety data (40). However, propranolol is safe in LQTS pregnancies (41) and may be preferred considering its higher efficacy in LQTS. If a non-selective β-blocker is used during pregnancy, consideration to switching to cardiac selective β-blocker like metoprolol in the third trimester should be given if childbirth by vaginal delivery is planned, as non-selective β-blockers can interfere with uterine contractions. Therapy with β-blockers is mostly well tolerated during pregnancy and the post-partum period though slightly lower fetal birth weights have been reported. Notably, β-blockers are secreted in breast milk, and rarely hypoglycemia and bradycardia may occur in breast-fed infants as a consequence of material β-blocker therapy.

## 4. Brugada syndrome

Brugada syndrome (BrS) is an IAS characterized by coved-type ST-segment elevation followed by a negative T-wave in the right precordial leads (V_1_-V_3_), either spontaneously or provoked by a sodium channel blocker, and increased susceptibility to SCD due to polymorphic ventricular tachycardia (VT) or VF (42). Over the past 25 years, 21 genes have been implicated in BrS (9) however, a recent evidence-based assessment of published literature found *SCN5A* to be the only gene definitely implicated causally in BrS (43). Pathogenic/likely pathogenic variants in the *SCN5A* gene are identified in around 20% of all BrS cases (44).

### 4.1. Sex-related differences in clinical phenotype

BrS primarily affects men; the BrS phenotype was recognized to be 8–10 times more common in Southeast Asian males than females (45). Because of this imbalance, there is paucity of studies analyzing the BrS phenotype and its consequences in females, and sex differences are underinvestigated. Registry data suggests that, women with BrS are more commonly asymptomatic, and on average 6 to 7 years older than men both at the time of diagnosis (49 vs. 43 years) and at the time of the first arrhythmic event (50 vs. 43 years) (46, 47). Female BrS patients less frequently have a spontaneous type 1 Brugada ECG pattern (22–41 vs. 36–69%) or ventricular arrhythmia inducibility at electrophysiology study (27–36 vs. 42–66%) (47, 48). Moreover, women are around 3–4-fold less likely to have syncope, aborted cardiac arrest and documented VF, than men with BrS (47, 49), except in the pediatric age group, where a spontaneous BrS ECG is associated with earlier arrhythmia onset–particularly, provoked by fever–in females (48). Therefore, males show a normal distribution of first arrhythmic event, while females show a bi-modal distribution (46). Whether there are sex-related differences in susceptibility to atrial fibrillation in BrS, remains to be investigated.

The higher incidence of Brugada EKG pattern in adult men vs. women suggests that testosterone plays an important role in ventricular repolarization. This was supported by the clinical observation that the coved-type ST-segment elevation disappeared after orchiectomy (50) or after androgen-deprivation therapy (51) in patients with asymptomatic Brugada syndrome who underwent treatment for prostate cancer. An effect of testosterone on ventricular repolarization is also suggested by the lower J-point amplitude in men with secondary hypogonadotropic hypogonadism (52). Additionally, a higher incidence of prostate cancer has been reported in men with BrS (53), which seems to correlate to higher testosterone levels in men with BrS compared to control men (45).

### 4.2. Sex-specific risk factors for arrhythmic events

Risk assessment in BrS is challenging, especially in women, since most studies report almost exclusively men, and only recently, female sex in BrS has been the focus of investigation. Analysis of large cohort registry reports indicates that women comprise less than one third (28%) of all BrS patients (46, 47, 54). Fragmented QRS and QRS prolongation (>120 ms) have been shown to be important risk factors for arrhythmias in women with BrS (HR 20.2 and 4.7, respectively), allowing for risk assessment beyond traditional risk factors, such as proband status, syncope and family history of SCD (HR 10.15, 6.8 and 69.4, respectively) (47). Interestingly, one study reported a higher prevalence of disease-causing *SCN5A* variants in asymptomatic female vs. male patients with BrS (27 vs. 21%), and a further difference in the prevalence of pathogenic variants in those with arrhythmic events exists (48% in females vs. 28% in males) (48), indicating a potential role for a genetic basis of BrS in arrhythmic risk. Furthermore, longer PR intervals have been reported as a marker of arrhythmias in female BrS patients (HR 1.03 per each ms of increase) (49). Intriguingly, atrioventricular conduction disturbances are frequently seen in BrS and highlight its overlap with cardiac conduction disease, both attributed to loss-of-function *SCN5A* variants in certain genetic forms of both conditions. Interestingly, there are reports of variants that show sex-dependent phenotypes, such as Gly1406Arg, which results in BrS in men and cardiac conduction disease in women (55).

Sinus node dysfunction occurs in nearly 1% of BrS patients (47). Certain familial BrS-associated *SCN5A* variants produce almost exclusively VF/SCD in men, but predominantly sinus node dysfunction and rarely VF/SCD in women (56). Since VF in BrS patients occurs almost exclusively during sleep, one hypothesis suggests arrhythmias in BrS might be provoked by bradycardia. In this case, concomitant sinus node disease could contribute to increased arrhythmogenesis. Studies have reported inconsistent findings regarding sinus node disease as an arrhythmia predictor in female BrS patients (47, 54).

The ventricular arrhythmias in patients with BrS have been linked to an arrhythmogenic substrate in the right ventricular outflow tract (57). Electrophysiological mapping studies of predominantly male patients with BrS have demonstrated that ajmaline exposes its extent and distribution, which is correlated with the degree of coved ST-elevation (58). Men with BrS have been shown to have a larger arrhythmogenic substrate as compared to women (59). Additionally, in both sexes, the arrhythmogenic substrate was larger in patients with disease-causing *SCN5A* variants than in those without.

### 4.3. Management

Current data suggests that most BrS patients will not experience life-threatening cardiac arrhythmias during their lifetime. As the risk of VF in asymptomatic patients with spontaneous BrS Type 1 ECG is rather low (≈1%/year), Brugada lifestyle precautions are sufficient for low risk patients (60), and include aggressive and prompt treatment of fever and avoiding of arrhythmia-provoking medications (for details, see www.brugadadrugs.org). Contrary to historical approach with liberal criteria for primary prevention ICD implantation, ICDs are currently recommended almost exclusively only in patients with a history of arrhythmic syncope and in cardiac arrest survivors (42). Given the lower risk of arrhythmias in women (2 vs. 5% within 5 years of diagnosis), women are less likely to require an ICD (20 vs. 34%) (47). Quinidine has been effective in preventing arrhythmias in symptomatic patients with BrS (61, 62), and should be considered in BrS patients with recurrent syncope or VT/VF, atrial fibrillation or in those who are reluctant to undergo ICD implantation (42). Instead of, studies have demonstrated that elimination of arrhythmogenic electrophysiological substrate in the RVOT epicardium by radiofrequency ablation results in ECG normalization and VT/VF non-inducibility (58), and reduced recurrent VT/VF episodes (63), suggesting that substrate-based radiofrequency ablation might be useful in selected patients with BrS, particularly those with multiple episodes of VT/VF. Yet, the knowledge regarding the arrhythmogenic substrate in females is much less studied given the small proportion of women in studies.

### 4.4. Pregnancy

There is limited data regarding pregnancy in BrS patients. One retrospective single-center study of 104 BrS women with 219 deliveries, reported no malignant arrhythmias during the pregnancy or peripartum period (64).

## 5. Catecholaminergic polymorphic ventricular tachycardia

Catecholaminergic polymorphic ventricular tachycardia (CPVT) is an IAS characterized by polymorphic ventricular arrhythmias provoked by high adrenergic tone (65). Pathogenic/likely pathogenic variants in the *RYR2* gene, encoding for the ryanodine receptor 2, underlie around 65% of all CPVT cases (autosomal dominant transmission), whereas variants in the *CASQ2* gene, encoding cardiac calsequestrin, are found in 2–5% of cases (mainly autosomal recessive inheritance) (26).

### 5.1. Sex-related differences in clinical phenotype

CPVT appears to equally affect both men and women (66). While initial reports indicated that males with *RYR2*-CPVT might be at a higher risk for SCD (67), recent data did not confirm this finding. The arrhythmic risk in *RYR2-*CPVT seems to be influenced by variant type and location. In a Canadian population, the founder *RYR2-*p.R420W variant showed earlier mortality in affected men compared to women (68).

A study investigating the circadian variation of arrhythmic events in pediatric patients with CPVT found that ventricular arrhythmias are more likely to occur in the afternoon and evening hours (69).

### 5.2. Sex-specific risk factors for arrhythmic events

Prior investigations reported no sex-specific risk factors for arrhythmias and SCD in CPVT. The general predictors for cardiac and fatal or near-fatal events are younger age at the time of diagnosis and absence of β-blocker therapy.

### 5.3. Management

Thus, far, the management is similar in men and women with CPVT. β-Blockers are the mainstay of therapy (26). Nadolol is more effective than β1-selective blockers. Flecainide in addition to β-blocker provides better prevention of exercise-induced arrhythmias in comparison to β-blocker therapy alone (70). LCSD may be useful for patients with drug intolerance or arrhythmias despite medical therapy. ICD is recommended in survivors of cardiac arrest with CPVT (26), but recent studies indicate these may increase the risk of fatal VF storms due to inappropriate ICD therapies, suggesting that strict adherence to medications alone may be superior (71, 72).

### 5.4. Pregnancy

Data suggests that the arrhythmic risk is not elevated during pregnancy and the postpartum period in patients with CPVT, yet events can occur in the absence of adequate β-blocker therapy unrelated to pregnancy (73). Therefore, continuous therapy with preferably non-selective β-blockers is indicated during pregnancy and the post-partum period to reduce the arrhythmic risk in women with CPVT (74).

## 6. Short QT syndrome

Short-QT syndrome (SQTS) is a very rare IAS characterized by shortened QT interval (QTc <330 ms) and increased susceptibility to atrial and malignant ventricular arrhythmias and SCD in the first decades of life (75). In the presence of a pathogenic variant in SQTS genes, family history of SQTS or SCD at age ≤40, or survival of a cardiac arrest due to VF in the absence of other heart disease, the diagnosis of SQTS can be made in the presence of a QTc <360 ms (26). SQTS1-3 are associated with gain-of-function variants in *KCNH2, KCNQ1*, and *KCNJ2*, respectively, whereas SQTS4-6 are caused by loss-of-function variants in *CACNA1C, CACNB2, CACNA2D1*, respectively (9). Most SQTS patients are diagnosed before the age of 40 years and are symptomatic with dizziness, syncope or SCD (75).

Nearly 70% of all patients diagnosed with SQTS are males, suggesting a sex-dependent penetrance (76). This could be explained by the longer resting baseline QTc in females than males; yet, no sex-associated difference in QTc intervals have been identified in SQTS patients (75, 76). Men have higher rates of syncope at first presentation (24 vs. 7%), but the rates of SCD are similar between the two sexes (24 vs. 25%) (76). A composite endpoint of life-threatening cardiac arrhythmias was observed more often in females (48%) than males with SQTS (28%), partly attributable to higher detection rate of VF in women given that all had ICDs in comparison to only 1/3 of men (76).

Given the high SCD risk in patients with SQTS, ICDs are the mainstay of therapy. ICD implantation is recommended in patients with SQTS and sustained VF, and may be considered in SQTS patients with a family history of SCD (26). Quinidine has been reported to successfully reduce the rate of arrhythmic events and might, thus, be used in asymptomatic SQTS patients (77). Sotalol may also have beneficial effect, but there is very limited data available supporting its efficacy (26).

## 7. Early repolarization pattern and early repolarization syndrome

The diagnosis of early repolarization syndrome (ERS) can be established in patients presenting with otherwise unexplained aborted cardiac arrest, documented VF or polymorphic VT in the presence of an early repolarization (ER) pattern in the inferior and/or lateral ECG leads (42). The ECG ER pattern is considered present if a J-wave (end QRS notch) or slur on the downslope of a prominent R wave can be identified with a J-point elevation of ≥ 0.1 mV in 2 or more contiguous leads of the 12-lead ECG, excluding leads V1–V3 (42). The J wave can manifest as an end QRS notch or as a slur on the downslope of a prominent R wave.

ER patterns, however, are commonly seen in the general population, with described rates ranging from 3 to 25% (78, 79). The ER pattern is more often found in young men (>70% of cases) than in women (80), with reduced prevalence and diminished sex differences with increasing age (81). A longitudinal follow-up study has shown that while the pattern was present in 25% of probands at age 25, it was only seen in 7% at age 45 (79). The ER pattern is more common in adolescents and athletes, as well as in African-Americans with a prevalence of up to 25% (79). Exercise training significantly increases the prevalence of ER pattern, which is particularly prevalent in athletes with bradycardia (82).

Although an ER pattern is typically a benign finding, some ECG features are associated with an increased risk for SCD. Presence of the ER pattern in inferior leads carries a higher risk than J-point abnormalities in the lateral ECG leads (83). Additional risk is conferred by J-wave elevations ≥0.2 mV, bradycardia and by a horizontal or downsloping morphology of the ST-segment (84). While sex-differences in terms of risk have not been reported, ethnicity may contribute to a higher arrhythmogenic risk with Caucasians being more susceptible (79, 80). In Asians or African Americans, ER pattern was not associated with an increased risk for tachyarrhythmias or SCD.

The presence of the ER pattern appears to increase the vulnerability for malignant arrhythmias, particularly in the setting of myocardial ischemia (82). Both the prevalence of ER pattern and the incidence of acute myocardial infarction are higher in men, which together may increase the risk of arrhythmic death in men, compared to women. Current evidence suggests that ER is a modifier of phenotype in other IAS. A meta-analysis of 5 studies and a total of 1,375 patients with BrS concluded that ER pattern is associated with a high risk of arrhythmic events in patients with BrS (85). In particular, BrS patients with inferolateral ER (global ER pattern) displayed the highest arrhythmic risk. In a study by Watanabe et al., ER pattern was associated with arrhythmic events in SQTS patients (86). In a small study of 52 CPVT patients, ER pattern was present in an unexpected large proportion (45%) of patients; it was more common in symptomatic patients and was associated with an increased frequency of syncope (87). Studies showed contradicting results regarding the clinical significance of ER in LQTS, with association with symptoms in an early report (88) and lack thereof in a more recent study (89).

Animal experiments point to differences in various ion current densities resulting in imbalances between epi- and endocardial layers as the electrophysiological basis underlying the ER pattern (90). Pathogenic variants in *KCNJ8* (ERS) and *KCND2* (in an atypical J wave syndrome) resulting in augmented K_ATP_ and I_to_ currents, respectively (gain-of-function), and variants in *CACNA2D1, CACNA1C*, and *CACNB2* resulting in an attenuated I_Ca_, and in *SCN5A* predicted to result in an attenuation of I_Na._, have been implicated in ERS (90). In particular, a larger I_to_ and ATP-sensitive current (I_KATP_) and a reduced *I*_Na_ and *I*_Ca, L_ in the epicardium vs. the endocardium lead to a greater net outward current early during the myocardial AP. Testosterone levels are significantly higher among men with an ER pattern compared to those without. Furthermore, the benign ER pattern with rapidly ascending ST-segment seems to be the pattern most closely associated with testosterone levels (91). As outward K^+^ currents are sensitive to testosterone levels, this may explain the higher prevalence of the ER pattern seen in men. A higher frequency of ER patterns in family members of probands with ER points, at least in part, toward a genetic basis (81, 92). While genetic findings are supportive of our current pathophysiological understanding (90), pathogenic variants have only been found in a minority of cases and our knowledge of disease heritability remains incomplete.

Management currently mainly depends on patient and family history; sex-specific differences in treatment algorithms have not been proposed (42). ICD implantation is recommended in ERS patients with documented VF. In the case of patient refusal, quinidine may be offered as an alternative therapy. ICD might also be considered in ERS patients with an arrhythmic syncope and high-risk ERS features or a strong family history of ERS-associated SCD at young age. If ERS patients present with electrical storm, both quinidine and isoproterenol have proven effective in stopping and preventing further arrhythmias (62). Both drugs reduce I_to_, thereby restoring transmural AP homogeneity.

ER patterns during pregnancy and the postpartum period have not been investigated.

## 8. Idiopathic ventricular fibrillation

Approximately 12% of sudden cardiac arrest survivors have no structural heart disease (93). Comprehensive diagnostic testing allows identification of subclinical structural cardiac abnormalities or IAS in up to half of these unexplained cardiac arrests (93, 94), whereas those with indeterminate cause of VF are referred to as idiopathic ventricular fibrillation (IVF) (95). IVF is estimated to account for 6.8% of all VFs (93), with prevalence increasing among older survivors. About 5–20% of IVF patients have a family history of SCD (96, 97), and in 9–17% genetic testing identifies potentially causal variants in genes mostly encoding cardiac ion channel subunits (98, 99), suggesting a significant overlap with the “concealed” forms of IAS. Because many IAS-related VFs were regarded as “IVF” prior to their discovery, advances in clinical recognition and genetic testing for IAS led to a decreased proportion of apparently unexplained cardiac arrests classified as IVF. The pathophysiology of IVF remains largely unclear. Few genes/variants have been clearly associated with IVF: *SCN5A*, Dutch *DPP6* risk haplotype, *CALM* genes, *RYR2*, and *IRX3* (99, 100).

IVF occurs in presumably healthy middle-aged individuals of either sex (average age ranging from 33 to 51 years) (101, 102). Clinical risk factors for IVF in the general population have not been established so far. A meta-analysis of 23 studies showed that males comprise about 70% of all IVF patients (101). Age at the time of the event appears to be comparable between men and women (103). Male sex, younger age, and presence of symptoms preceding the index event seem to be associated with multiple appropriate ICD shocks (103). Male patients are also more likely to receive a specific diagnosis during follow-up (103). Among carriers of the *DPP6* haplotype, men show higher rates of VF and lower survival than women (63 vs. 83 years) (104). The mechanisms underlying these disparities remain to be investigated.

About 30% of IVF patients experience recurrent VF within 5 years (101). Therefore, an ICD is recommended for secondary prevention of SCD in all IVF patients (26). In *DPP6* haplotype-positive individuals, the estimated risk of SCD is close to 50% by the age of 60 (104). Primary prevention ICD implantation or therapy with quinidine can be considered in this population. A subgroup of IVF patients has short-coupled premature ventricular complexes (scPVCs) from the specialized conduction system that can trigger so-called short-coupled TdP or VF (105). In those, mapping and ablation of scPVCs can prevent recurrent VFs and ICD shocks (105).

Complete evaluation of IVF patients including onset on ECG or ICD interrogation is critical for guiding clinical evaluation and cascade genetic testing in affected families, since relatives of survivors might be at similar risk for SCD (106). When a genetic origin is detected, functional characterization of the causal variant(s) might provide insight into the disease pathophysiology and help guide therapy for a selected subset of patients.

## 9. Progressive cardiac conduction disease

PCCD is a hereditary disease characterized by progressive and unexplained cardiac impulse conduction delay, with ensuing predisposition to complete AV block, syncope, and SCD (107). Familial forms of PCCD in the absence of structural heart disease are typically caused by variants in cardiac ion channel genes (108), with variants in *SCN5A* being the most common genetic substrate in isolated PCCD (108). However, the proportion of cases attributable to *SCN5A* remains unclear due to limited number of reports.

Sex-related differences in PCCD have not been investigated. As in other *SCN5A-*mediated diseases, the disease seems to be more common among men, but robust data is lacking. Because most reports on PCCD are limited to individual cases or families, data on risk during different reproductive phases does not exist.

## 10. Familial ST-depression syndrome

Familial ST-depression syndrome is an inherited disease characterized by persistent, non-ischemic concave ST-depressions in multiple leads, associated with an increased risk of atrial fibrillation, SCD and (in older persons) some degree of left ventricular dysfunction (109). Familial ST-depression syndrome is diagnosed in the presence of (1) unexplained concave-upward ST depression in at least 7 leads, 90 ms after J point, (2) ST-elevation in lead aVR > 0.1 mV, (3) ECG findings persistent over time, and (4) autosomal dominant pattern of inheritance. The ST-segment depression develops in prepuberty, progress slowly, and are most pronounced in leads V_4_, V_5_, and II. Evaluation of the pedigrees indicated an autosomal dominant pattern of inheritance in all affected families. The genetic background of this syndrome is yet to be identified, as gene panels have not revealed a causative variant in known inherited heart disease associated genes. Based on the limited data available, the onset of complications does not appear to correlate with age in affected individuals.

The limited available data suggests strong sex-related differences in the arrhythmic phenotype of familial ST-segment depression syndrome. In a cohort of 40 individuals (43% men) from 14 apparently unrelated Danish families with ≥2 affected members, over a mean follow-up of 9 years, syncope occurred in 20%, atrial fibrillation was observed in 10 patients (25%, 7 men), (aborted) SCD in 5 patients (13%; 4 men), and left ventricular systolic dysfunction occurred in 10 patients (25%, 7 men) (110). The occurrence of ventricular arrhythmia and left-ventricular systolic dysfunction almost exclusively in men suggests a sex-specific natural history. The pathophysiological basis of these sex-specific events remains to be investigated.

## 11. Important considerations regarding pregnancy and the peripartum period

Pregnancy and the peripartum period are both associated with significant neurohumoral changes, which should be taken into account in patients with IAS. In particular, stressors, by increasing sympathetic tone, and drugs can provoke torsade de pointes and polymorphic VT, leading to syncope, seizures, or SCD in these patients (111). The balanced approach to pregnant women with IAS should consider the maternal and fetal risks related to the disease as well as antiarrhythmics used. Identification of known high-risk features, avoidance of specific arrhythmia triggers, preventive therapy when needed, and neonatal screening when available, are key to optimal medical care. Generally, children are at relatively low risk for arrhythmias during the first year of life (except those with *de novo* genetic disease); nevertheless, they should be screened appropriately given that 5–10% of all sudden infant death syndrome cases are attributed to IAS (112).

Generally, unassisted vaginal delivery may be performed in women with IAS (1). However, the delivery plan should be individualized according to the maternal risk profile, considering the history of any relevant arrhythmias. In high-risk patients, availability of a cardiologist or a cardiac electrophysiologist and use of maternal cardiac telemetry during labor are recommended. The choice of anesthetics should be made carefully taking into account the list of proarrhythmic medications to avoid drug-induced adverse reactions (e.g., drugs that interfere with cardiac repolarization and prolong the QT interval are strongly discouraged in patients with LQTS) (111). Limited evidence suggests, single bolus propofol may be used for induction of anesthesia in patients with BrS, but higher doses of propofol and longer infusions may potentially be associated with significant risk of arrhythmias and are presently not advisable (113).

Since IAS are rare diseases, only limited evidence exists regarding the outcome of pregnancies in women with IAS. Overall, arrhythmias seem to be very rare during labor. In patients with LQT1, LQT2, and CPVT, arrhythmic events are more likely to be provoked by increased heart rates, which are typically seen in the active pushing phase of labor. Notably, heart rate increases significantly in patients receiving intravenous oxytocin. Oxytocin also prolongs cardiac repolarization and may lead to TdP in those with LQTS (114) and thus, should be used with caution during labor.

## 12. Conclusions and future perspectives

A growing body of literature demonstrates substantial biological sex-related differences in the incidence and clinical phenotype of various IAS, most notably the higher prevalence of QT prolongation in women and male preponderance of BrS. Despite multiple basic and clinical studies showing an effect of sex hormones on outcomes in patients with IAS, many clinically relevant questions remain to be addressed. Thus, a precision medicine approach including the consideration of sex-specific characteristics should be integrated in the care of IAS patients. Further characterization and awareness of differences in symptom presentation, disease progression, outcomes and treatment response present new opportunities for improving patient care and for paving the way for precision medicine.

## Author contributions

BA and AB manuscript drafting and review. All authors contributed to the article and approved the submitted version.
